# Infantile Diarrhœa

**Published:** 1891-09

**Authors:** 


### INFANTILE DIARRHŒA.

It is more than probable—and this theory is finding favor among the best thinkers in the profession—that the fermentive changes following general enfeeblement produced by excessive heat, engenders tyrotoxicon. Frequently, after emesis and diarrhœa are under perfect control, patients succumb to peculiar brain lesions which appear to be toxemic.

It is certainly rational treatment to arrest abnormal fermentive changes and render innocuous the toxic products of these changes. Various means of retarding or stopping degenerative change have been proposed and practiced, many of which have some merit; yet it is not sufficient, in many cases, to prevent further fermentation, but it is also requisite to render inert or insoluble toxic elements forming or already formed. We have used cupri arsenitis 1-100 grain in four ounces of water, giving teaspoonful doses every ten or fifteen minutes for the first hour, and hourly afterward till vomiting and diarrhoea were under complete control. This treatment, in connection with that mentioned in the July number of the *Journal*, has afforded us great satisfaction. It may be added in passing, that this treatment is very efficient in dysentery.

Dr. Luff (*Lancet*) speaks very warmly of the power of the soluble biniodide of mercury to destroy or render insoluble the ptomaine, tyrotoxicon, and stop abnormal fermentive changes. He administers one-fiftieth of a grain of biniodide of mercury for a child up to the sixth month. His review of eighty cases shows that diarrhoea stopped within three days in seventy two, within four days in five, and no case lasted over seven days.—*Indiana Med. Journal.*

